# Static axial overloading primes lumbar caprine intervertebral discs for posterior herniation

**DOI:** 10.1371/journal.pone.0174278

**Published:** 2017-04-06

**Authors:** Cornelis P. L. Paul, Magda de Graaf, Arno Bisschop, Roderick M. Holewijn, Peter M. van de Ven, Barend J. van Royen, Margriet G. Mullender, Theodoor H. Smit, Marco N. Helder

**Affiliations:** 1 Department of Orthopaedic Surgery, VU University Medical Center, Amsterdam Movement Sciences, The Netherlands; 2 Department of Epidemiology and Biostatistics, VU University Medical Center, Amsterdam, The Netherlands; 3 Department of Plastic, Reconstructive and Hand Surgery, VU University Medical Center, Amsterdam, The Netherlands; 4 Department of Anatomy, Embryology and Physiology, Academic Medical Center, University of Amsterdam, Amsterdam, The Netherlands; 5 Department of Oral and Maxillofacial Surgery, VU University Medical Center, Amsterdam, The Netherlands; Seoul National University College of Medicine, REPUBLIC OF KOREA

## Abstract

**Introduction:**

Lumbar hernias occur mostly in the posterolateral region of IVDs and mechanical loading is an important risk factor. Studies show that dynamic and static overloading affect the nucleus and annulus of the IVD differently. We hypothesize there is also variance in the effect of overloading on the IVD’s anterior, lateral and posterior annulus, which could explain the predilection of herniations in the posterolateral region. We assessed the regional mechanical and cellular responses of lumbar caprine discs to dynamic and static overloading.

**Material and methods:**

IVDs (n = 125) were cultured in a bioreactor and subjected to simulated-physiological loading (SPL), high dynamic (HD), or high static (HS) overloading. The effect of loading was determined in five disc regions: nucleus, inner-annulus and anterior, lateral and posterior outer-annulus. IVD height loss and external pressure transfer during loading were measured, cell viability was mapped and quantified, and matrix integrity was assessed.

**Results:**

During culture, overloaded IVDs lost a significant amount of height, yet the distribution of axial pressure remained unchanged. HD loading caused cell death and disruption of matrix in all IVD regions, whereas HS loading particularly affected cell viability and matrix integrity in the posterior region of the outer annulus.

**Conclusion:**

Axial overloading is detrimental to the lumbar IVD. Static overloading affects the posterior annulus more strongly, while the nucleus is relatively spared. Hence, static overloading predisposes the disc for posterior herniation. These findings could have implications for working conditions, in particular of sedentary occupations, and the design of interventions aimed at prevention and treatment of early intervertebral disc degeneration.

## Introduction

Lumbar disc herniation (LDH) and disc protrusion are phenomena occurring with degenerative disc disease (DDD), often causing acute exacerbation of pain symptoms [1–3]. Disc degeneration usually begins in the third decade of life and peak incidence of LDH is in the fourth and fifth decade [4]. The caudal segments are affected more commonly (i.e. L5-S1 more than L4-5) and the posterolateral corner of the lumbar intervertebral disc (IVD) is the most common site to herniate [5]. Besides genetic factors and extrinsic factors like smoking and obesity, spinal loading conditions have been identified as risk factors for developing both disc degeneration and a lumbar hernia [6–9]. In fact, a recent observational study in a general adult population endorsed the finding of Wilder *et al*. [10] that physical loading and sitting hours (static axial load on the spine) are the most important risk factors for developing a lumbar hernia [11].

It is a common misconception that hernia’s arise only in aged or degenerated IVDs. As Lama *et al*. show in a recent clinical study on human herniated discs of working age adults, herniated discs needing surgery had only mild to moderate degeneration on the Pfirmann score, and the herniated nucleus pulposus (NP) tissue did not show significant loss of proteoglycans or water compared to controls [12]. This implies that, at least in a subtype of hernia’s and/or subgroup of patients, hernia’s will occur as a result of a more focal degenerative process in the annulus, which combined with a relatively healthy state of the NP will lead to fissuring of the annulus and extrusion of the pressurized NP tissue.

The shape of the disc and the presents of the posterior longitudinal ligament have been described as factors contributing to the posterolateral predilection of hernia’s [13]. The collective works of Holzapfel, Adams, Hutton and McNally describe the annular mechanical function of human IVDs and the regional internal stresses under various loading conditions in detail and provide some biomechanical rational for posterior herniation during short-term complex (i.e. axial load and flexion, flexion-torsion) and near-failure loading conditions [14–21]. Whether the described internal asymmetry of pressure distribution has any effect on the external pressure distribution, shape or function of the disc (increased wedging and/or higher peak pressure transferred through the posterior IVD), or biological consequences on IVD cells and matrix in the posterior annulus during prolonged periods of loading has not yet been studied. Furthermore, it does not explain why many herniations in patients occur without a clear inciting moment such as heavy lifting or the combination of flexion and torsion, but during seemingly arbitrary loading conditions [22]. Taken together, much remains uncertain as to why some people develop a hernia while others have “uncomplicated” ageing and degeneration of their lumbar IVDs.

Studies on human and various types of animal (lumbar) IVDs have shown a correlation between high mechanical forces applied to the disc, and degenerative changes [23–28]. In recently published work from our group with the Loaded Disc Culture System (LDCS; a bioreactor for whole organ culture of large species IVDs) we studied the influence (mechanical and biological responds) of caprine IVDs to various types of loading conditions during ex vivo culture [29]. We found that both high static and high dynamic axial overloading had negative effects on disc cells and matrix. Interestingly, where dynamic overloading was detrimental to both the nucleus and the annulus region, static overloading was especially harmful for the annulus of the IVD [30]. This could provide a rational for why relatively healthy discs can herniate. However, we have limited information on the possible difference in effect to the anterior, lateral and posterior outer annulus, which could explain the posterolateral predilection of hernia’s.

Therefore, we conducted a series of experiments with the LDCS to investigate the influence of strictly axial dynamic or static mechanical overloading on the various regions of the IVD. We address the question of possible location dependent effects by analyzing the regional biomechanical response (height loss and pressure distribution) in the disc to axial loads during culture and how this influences the cells and matrix in the various disc regions over time. We hypothesize that strictly axial static overloading will affect the cells and matrix in the posterolateral region more strongly than in other regions of the intervertebral disc.

## Material & methods

### IVD specimens

Thirty lumbar spines from healthy skeletally mature (3–5 year-old) goats (Capra aegagrus hircus, sub breed Dutch white milk goat) were used for the experiments. The lumbar spine and IVD of this specific goat species has been extensively studied and has been shown to closely resemble the human IVD with respect to biomechanical properties, cell population and matrix composition [29,31–33]. Cadaver caprine spines used in the current study were obtained after slaughter from an abattoir in The Netherlands (Firma vd Horst, Maarssen, N 52° 09.008' E 005° 01.327') and as we use remnants of slaughter animals no approval of an ethical committee is required. Within 3 hours after slaughter, the exterior of lumbar spines were sterilized using a medical grade iodide-alcohol solution prior to dissection under sterile conditions of the lumbar IVDs (Th13-L6; n = 180 total). IVDs with adjacent cartilaginous endplates were dissected using an oscillating surgical saw and closely inspected to detect any signs of disease or degeneration (and excluded if any anomalies were found). The discs are dissected by sawing in two parallel planes as close as possible to the proximal and distal endplates, preserving the cartilaginous endplate but removing all excess bone tissue. The sawing planes are perpendicular to the central axis of the individual motion segment. IVDs were cleaned with sterile gauze to remove any debris, blood and muscle or ligament tissue (especially remainders of the posterior longitudinal ligament) and placed in a 6-wells plate with culture medium prior to placement in the LDCS. From each spine, IVDs (Th13-L1 and/or L5-L6; 35 IVDs total) were used as baseline reference (day 0) for the parameters measured. IVDs were cultured and loaded in a bioreactor (the LDCS; see below) for 14 days. Separate culture experiments were performed for biomechanical measurements, and histology and quantitative cytology.

### IVD culture and loading

Lumbar IVDs were cultured for 14 days in individual culture chambers in the previously described Loaded Disc Culture System (LDCS) [29], which is housed in an incubator at 37°C, 95% humidity, and 5% CO2. Discs were cultured in standard DMEM (Gibco, Paisley, UK) with 10% FBS (HyClone, Logan, UT), 4.5g/L glucose (Merck, Darmstadt, Germany), 50 μg/ml ascorbate-2-phosphate (Sigma Aldrich, St. Louis, MO), 25 mmol/L HEPES buffer (Invitrogen), 10,000 u/ml penicillin, 250 μg/L streptomycin, 50 μgr/mL gentamicin and 1.5 μgr/mL amphoterizin B (all from Gibco).

Mechanical loading of the IVDs was strictly axial. Loading magnitudes and frequency were derived from in vivo pressure measurements in a lumbar segment of a goat during different activities (e.g. lying down, walking and jumping on a haystack) [34]. For standardization, all discs were subjected to a sinusoidal preload (LDL); ~0.1 MPa; 1Hz) during the first 8 hours of culture.

**Fig 1:**
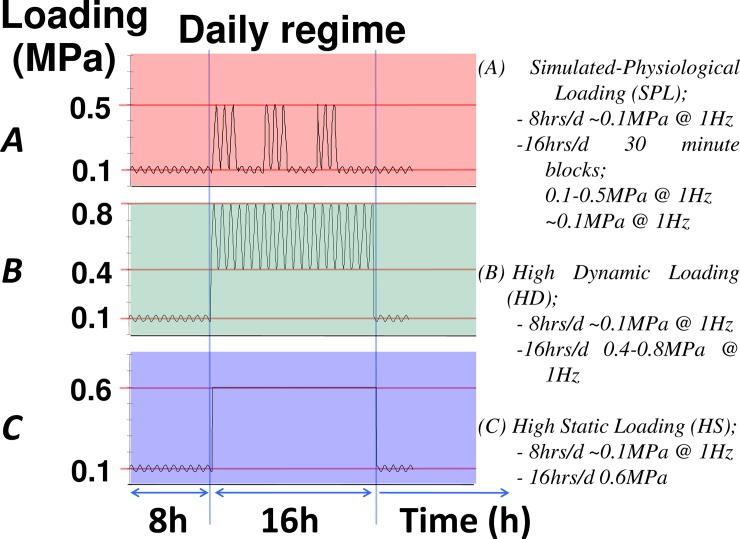
Scheme of the three daily loading regimes. Shown on the Y-axis is the axial load (MPa) as applied on the IVDs. Shown in the upper panel (A, red) is the simulated-physiological loading regime, in the middle panel (B, green) the high dynamic loading regime, and the lower panel (C, blue) the high static loading regime. All regimes start with 8 hours of low dynamic load around 0.1 MPa, after which a 16 hour loading regime is applied as indicated in the caption.

IVDs were subjected to either a high dynamic or a high static daily loading regime (Fig 1):

1. High dynamic loading (HD): sinusoidal load (1Hz) alternating in magnitude from 0.4 to 0.8 MPa for 16 hours per day, followed by 8 hours of LDL.
2. High static loading (HS): static load of 0.6 MPa during 16 hours/day, followed by 8 hours of LDL.

Both loading regimes finished with an 8 hours interval of LDL loading. In previous reports we have shown that native caprine disc properties can be maintained over 21 days in LDCS culture, when IVDs are loaded with a simulated-physiological loading [29], whereas the above described high dynamic and static loading have detrimental effects on caprine discs [30].

### IVD geometry and height loss

After dissection from the spine a baseline measurement of the maximum width, mid-sagittal depth, and height of each IVD was taken with a calliper. Contours of both proximal and distal endplate were traced on paper at baseline. Tracings were digitized (Canon MP620, 2400dpi, Tokyo, Japan) and used to calculate the exact surface of the endplate (mm^2^) using Image-J software (Rasband WS. ImageJ, National Institutes of Health, Bethesda, Maryland, USA, http://rsb.info.nih.gov/ij/; 2007–2015), and verified with Tekscan contact area measurements. Prior to every pressure mapping experiment (every 48hrs), disc height (anterior, mid-sagittal and posterior) was measured again, to assess disc height loss

### Pressure mapping

Pressure mapping was performed at baseline (day 0 measurement) and subsequently every 48 hours (after the loading phase) during the 14-day culture period. Contact pressure distribution was measured using a thin film pressure-sensitive system (I-scan 5051, Tekscan Inc., Boston, MA, https://www.tekscan.com/products-solutions/pressure-mapping-sensors/5051). The sensor was pre-conditioned according to manufacturer’s guidelines and calibrated according to recommendations from Brimacombe *et al*. [35].

For pressure mapping, IVDs were taken out of the culture chamber and placed with the proximal endplate on the sensor surface, which in turn was placed on a 15 mm thick glass plate. A static load of 30 kg (300N) was applied via a universal hinge to ensure axial application of the load, regardless of potential uneven IVD height loss. The pressure distribution was recorded after 2 minutes with a sampling frequency of 10 Hz. Average values of 10 consecutive frames (1s) were used for analysis.

Raw sensor output data from each time point was analysed with a custom MATLAB program (MathWorks, Natick, MA). Contact area (mm^2^) of the endplate impression on the sensor was derived from the number of sensels (sensor pixels) with a signal above threshold value (16 kPa) and the known sensel dimensions. The disc area was divided in an anterior to posterior region with equal contact areas. Mean pressure per region was calculated from the sum of values per sensel in a specific CA, divided by that specific contact area. The distribution of pressure over the anterior and posterior region was compared at each time-point between experimental groups and within each group over the culture period.

### Cell viability

Transverse cryosections (10μm) were prepared of IVDs from all groups at day 0 (baseline control discs from Th13-L1 and/or L5-L6) and after the 14-day culture (culture experiment discs from L1-L2 to L4-L5). Sections were stained with a fluorescent live/dead staining (Cell Tracker Green and Propidium Iodide) as described previously [29]. On average one hundred images (sized 1048×1342 pixels) were shot using an automated rig on the hystomorphometric microscope between programmed coordinates at the borders of the IVD cryosection at 10× magnification (area≈1mm^2^) using fluorescent light on an inverted microscope (Leica DM6000, Wetzlar, Germany). The individual images were automatically stitched together by the Leica software to compile to a complete image of every half IVD. These stitched images were used to identify the five distinct regions of the IVD (1. nucleus; 2. inner annulus; 3. anterior outer annulus; 4. lateral outer annulus; 5. posterior outer annulus) and to count the live (green) and dead (red) cells. The percentage of live cells (100% (#live cells/ #total cells)) was determined using 10 images per region for each IVD. Co-labeled cells were excluded from the analysis of cell viability. A fresh (day 0) IVD was used as positive control. As a negative control, a thoracic IVD, which underwent a freeze-thawing cycle three times prior to staining, was used.

### Histology

Directly after spinal dissection (baseline controls), or after the 14-day culture in the LDCS, IVDs were divided in halves; the left half was used for sagittal sectioning (a 3mm thick paramidsagittal tissue slices) and the right half was used for transverse sectioning (full thickness of IVD). Specimens were fixed in formaldehyde, decalcified using Kristensen’s solution (formic acid decalcifier buffered with formate), embedded in paraffin and 3-μm sections were cut with a microtome. Sections were stained with a standard H&E, alcian-blue staining and safranin-O staining. Histological classification of degeneration was performed according to the Rutges scale [36]. Baseline controls (day 0 IVDs) were used for region definition, sections of the SPL loaded IVDs were used as controls for the overloaded experimental groups.

Immunohistochemistry was performed using the fully automated Benchmark Ultra device (Ventana Medical Systems Inc., the Roche Group, Tucson, AZ, USA) with Optiview detective method. Cell apoptosis was detected using a cleaved Caspase-3 antibody (Cell Signaling Technology Inc. Danvers, MA, USA; rabbit poly clone, 9661-L, 1/100 dilution, 32 min incubation, 24 min retrieval). As a negative control we omitted the primary antibody, human tissue sections (healthy skin and cancerous trachea) were used for as positive controls.

### Statistical analysis

All measurements were analysed using linear mixed models. Experimental outcome parameters were included as dependent variables in the models. A random effect for each goat and IVD combination was included in the models. The models investigating the impact of the loading conditions over time included a fixed effect for loading condition and time point and the interaction between time and loading condition. Post-hoc testing with Bonferroni correction was used to compare mean outcomes between loading conditions separately for each time point. Pressure distribution over time was compared between regions in a similar way separately for each loading condition. Mean cell viability was compared between baseline measurements and the 14-day follow-up measurements after different loading conditions using a mixed model with a fixed effect for group (baseline, SPL, High Dynamic or High Statistic) followed by post-hoc pairwise comparisons of estimated means with Bonferroni correction. This analysis was done separately for each of the five disc regions. *P* values <0.05 were considered significant. Descriptive data are presented as means ±SD. Statistical analysis was performed using SPSS 20 software (IBM Corporation, Armonk, NY, USA).

## Results

### Baseline IVD geometry and region definition

The baseline IVD geometry of the experimental discs (L1–2 to L5–6) are given in Table 1. There were no significant differences in any dimension at baseline (e.g. proximal IVDs being slightly narrower and deeper than their distal counterpart as in human discs); neither were there any significant differences in IVD dimensions at baseline between the experimental groups.

**Table 1:** Baseline IVD geometry (mm).

Disc level	Max. width	Midsag. depth	Total height	Surface (mm^2^)
L1-L2	25.6 ± 1.6	18.9 ± 1.1	7.9 ± 1.1	380.6 ± 32.6
L2-L3	26.1 ± 1.8	18.8 ± 1.0	7.1 ± 1.0	387.2 ± 45.4
L3-L4	26.7 ± 1.9	19.3 ± 1.0	8.2 ± 1.4	404.9 ± 48.4
L4-L5	27.1 ± 1.3	17.9 ± 0.8	7.3 ± 1.6	382.4 ± 28.1

Table 1. Average measurements of baseline dimensions of the lumbar IVDs used for the loading experiments (L1-L2 through L4-L5, n = 120). All dimensions are given in millimeters +/- standard deviations, respectively maximum width, midsagital depth, total height of the endplate-IVD-endplate motion-segment, and the total surface (mm^2^ ±SD). No significant difference were found (p≥0.05) when comparing measurements between disc levels, nor were there any differences in the allocated discs per experimental group.

**Fig 2:**
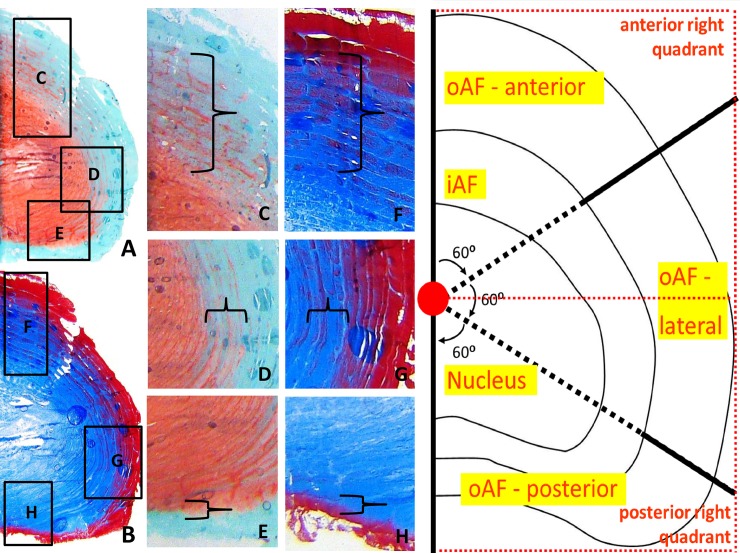
IVD region definition. The left panel shows representative images (2.5 × magnification) of transverse histological sections of a right half of an IVD at baseline (A; safranin-O, B; Masson’s trichrome). Images C through E show detailed images (10 × magnification) of the safranin-O staining, in which proteoglycans (primarily in the nucleus) are stained red and a fast-green counterstaining visualizes the rest of the IVDs matrix. Images F through H show detailed images of the Masson’s Trichrome staining, in which the dense matrix of the nucleus (primarily type 2 collagen and GAGs) is stained blue, whereas the more porous structure of the annulus (primarily collagen type 1) is stained red. Images C and F show the anterior region of the outer annulus of the disc with its distinct broad transition zone (accolade); Panels D and G show the lateral annulus region with a narrower transition zone (accolade); Panels E and H show a detail of the posterior region of the disc with the distinct sharp demarcation between the nucleus and outer annulus region (accolade).The right panel is a schematic drawing of the right half of a caprine lumbar IVD. We can observe that the midsagittal line is drawn from the upmost anterior edge of the IVD to the bottommost posterior edge of the IVD. The red dot in the middle of this line represents the geometrical center of the IVD, from which the two black semi-dotted 60 degree lines are drawn to distinguish the anterior (0°-60°), lateral (60°-120°) and posterior part (120°-180°) of the outer annulus. Also drawn from the center is a red dotted line to distinguish the upper right en lower right quadrant as used for the Tekscan analyses. The surfaces of the various regions did not differ significantly.

On the baseline histological slides (day 0) five distinct IVD regions were defined. In the right panel in Fig 2, a scheme is drawn on the right half of a caprine lumbar IVD. We can observe that the midsagittal line is drawn from the uppermost anterior edge of the IVD to the mid-posterior edge of the IVD. The red dot in the middle of this line represents the geometrical center of the IVD. Also drawn from the center is a red dotted line to distinguish the upper right en lower right quadrant as used for the Tekscan analyses. The surfaces of the various regions did not differ significantly. The five region can be distinguished: nucleus (NP), the inner-annulus (iAF) and three outer annulus (oAF) regions: anterior (oAF-a; 0°-60°), lateral (oAF-l; 61°-120°) and posterior (oAF-p; 121°-180°) region (Fig 2, right panel).

Nucleus, inner- and outer-annulus were distinguished by their gross morphological structure, together with their appearance on histological Safranin-O and Masson’s Trichrome stainings (Fig 2, left panel). On the transverse sections, the nucleus region had the most intense red staining (Safranin-O) and no lamellar structure (Masson’s trichrome). The inner-annulus had some red staining and some lamellar organization. The outer-annulus had the least red staining on Safranin-O and clearly defined lamellae (Fig 2, middle panel). Morphological appearance on all histological sections showed the annulus lamellae in the anterior section to be broad with a bricklike pattern, narrowing in the lateral section and with a thin, dense and more parallel pattern in the posterior section (Fig 2, middle panel).

### IVD height loss during culture

**Fig 3:**
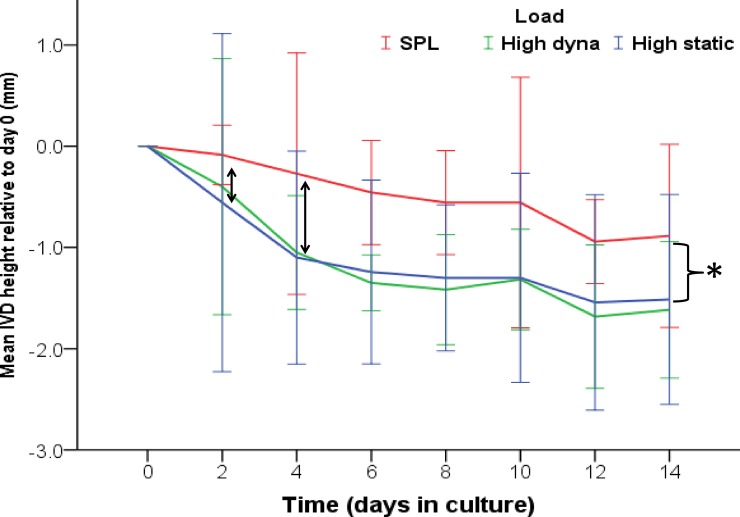
Graph of IVD height loss during the 14 day culture experiments. Height loss of IVDs (mean height loss (mm ± SD), relative to baseline (day 0) are shown for the three experimental groups (SPL (red), HD (green) and HS (blue)) at each time point (every 48 hrs) during culture in the LDCS. Arrows at day 2 and 4 indicate the quickly increasing difference in disc height between the overloaded IVDs and the SPL loaded discs. The accolade shows the significant difference (* indicates *p*<0.05) in height after 14 days of culture and loading between the SPL loaded IVDs and the overloaded discs.

We found no difference in disc height between the anterior and posterior side of the IVDs after axial loading (no wedging occurred); therefore, overall mid-sagittal height was used in analyses. We can observe in the graph (Fig 3) that between day 0 and day 4 in the overloaded groups, on average, discs suffered more height loss than their SPL counterparts, although it is clear from the large standard deviations, within load groups there are still large inter-individual variations. After 14 days of culture and loading, IVDs have significantly lost more height (measured at the end of an LDL resting phase) in the overloaded groups when compared to the SPL group (SPL mean height loss 0.89mm (±0.45); HD 1.62mm (±0.45; *p* = 0.008) and HS 1.51mm (±0.52; *p* = 0.032). Between the high dynamic and high static overloaded IVDs there was no significant difference at any time-point.

### Pressure distribution during culture

To validate the Tekscan output we cross-checked contact area measurements with the Image-J calculation and pressure measurements with LDCS output. Measures correlated with an r^2^-value of 0.94 and a systematic under representation of IVD size in the Tekscan values of 15–20%. Pressure distribution mapping over the proximal endplate during a static 30kg (300N) axial showed that pressure values were always highest in the centre of the disc (nucleus region). During dynamic loading, pressure shifted from the centre towards the periphery of the IVD.

**Fig 4:**
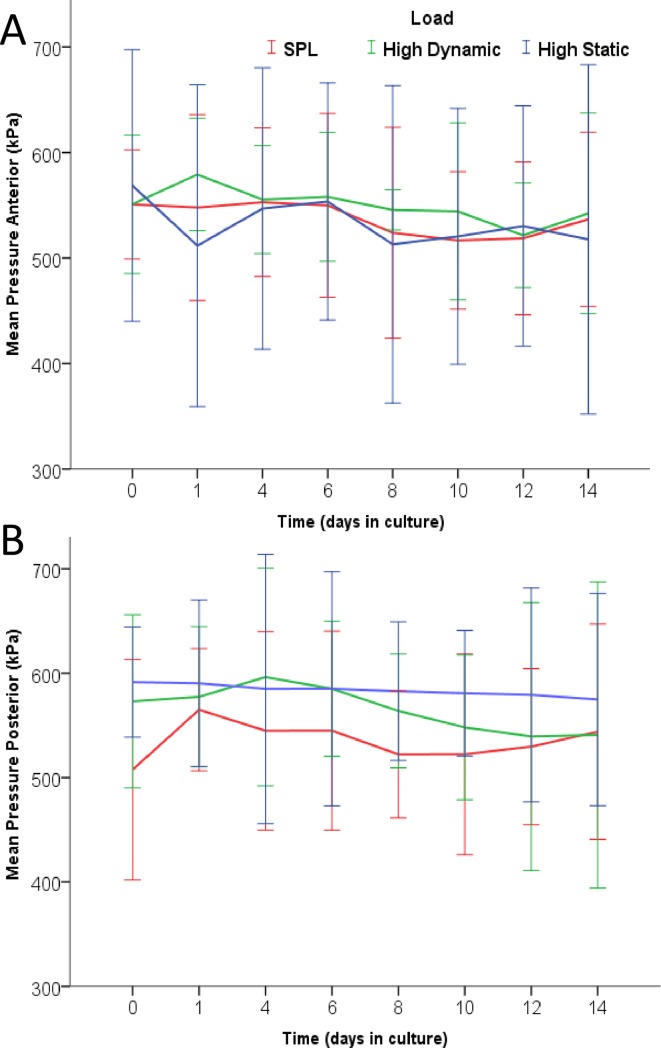
Pressure distribution during culture. Graphs show the mean pressure (kPa; mean ±SD) over the proximal cartilaginous endplate for the anterior (a) and posterior (b) disc region during a static axial load (300N). Measures are shown per experimental group; SPL (red), HD (green) and HS (blue), at every time point (48 hrs) during culture in the LDCS. Pressure measurements on the IVDs were performed after the 16 hour loading phase of each experimental group.

In Fig 4 the mean pressures over the anterior (Fig 4A) and posterior (Fig 4B) side of the endplate during the 14-day culture experiment are shown. For the anterior region we observed that the mean pressure did not differ between the loading groups at any time-point during culture (Fig 4A). For the posterior side, we can observe the line of the static group being slightly higher overall compared to the other groups, however this is not statistically significant during the 14 day culture period (Fig 4B).

When comparing the pressure distribution over the 14-day culture period within each load group, distribution over the contact area of the IVD (anterior, lateral or posterior) was not found to be different at the different time-points. Neither were there any statistical differences in pressures measured between baseline (day 0) and any other time point during culture between any disc regions within any load group.

### Cell morphology, viability and apoptosis after culture

**Fig 5:**
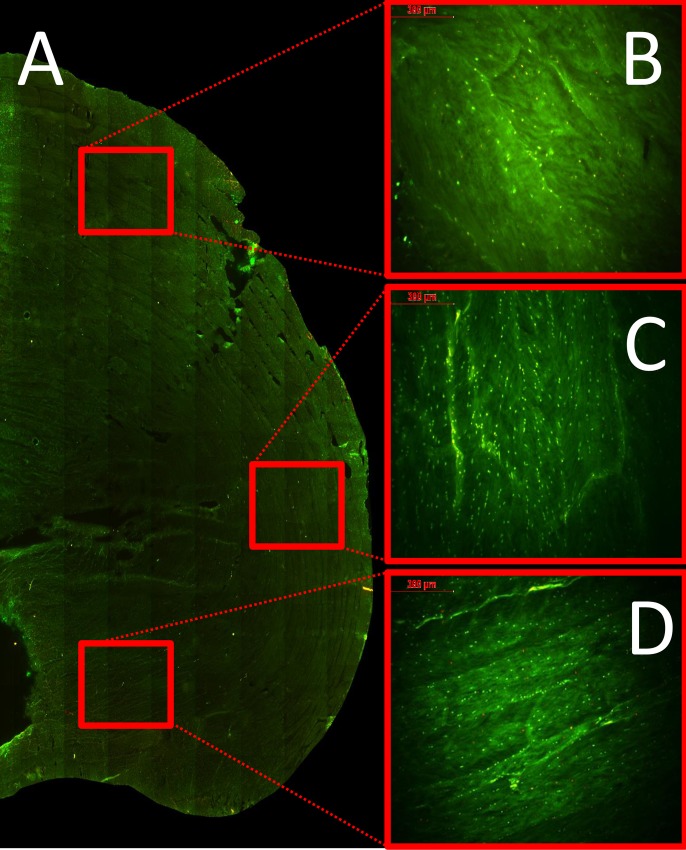
Fluorescent cell viability mapping in the IVDs. A representative stitched image (baseline control) of fluorescent Cell Tracker Green (life cells) and Propidium Iodide (dead cells) labelled cells in a half IVD (a); detailed images of the three distinct outer annulus regions are shown in images b (anterior), c (lateral) and d (posterior).

Fig 5 shows a representative stitched image of a SPL loaded half IVD (Fig 5A, 2,5x magnification) stained with the fluorescent live/dead staining. Detailed images (10x magnification) of the anterior (Fig 5B), lateral (Fig 5C) and posterior (Fig 5D) outer annulus are shown in the right panel. We can observe a difference in appearance of the lamellar structure of the respective annulus regions. Gradually changing from a brick-like pattern with broad header and stretcher lamellae in the anterior section (Fig 5B), towards a more rope like structure with narrow lamellae alongside each other in the posterior section (Fig 5D). Also, the fluorescent staining reveals the cell morphology and distribution differences in the IVD regions; the nucleus and anterior inner- and outer annulus having fairly round and evenly distributed cells, whereas in the lateral and posterior part of the annulus cells are more elongated and clustered between and at cross points in the lamellae. In the overloaded groups we could observe that the PI positive stained cells versus total cell ratio was approximately 1:4 in all disc regions in the dynamically overloaded IVDs and between 1:5 and 1:2 for respectively the anterior and posterior annulus in the statically overloaded IVDs (Fig 5A).

**Fig 6:**
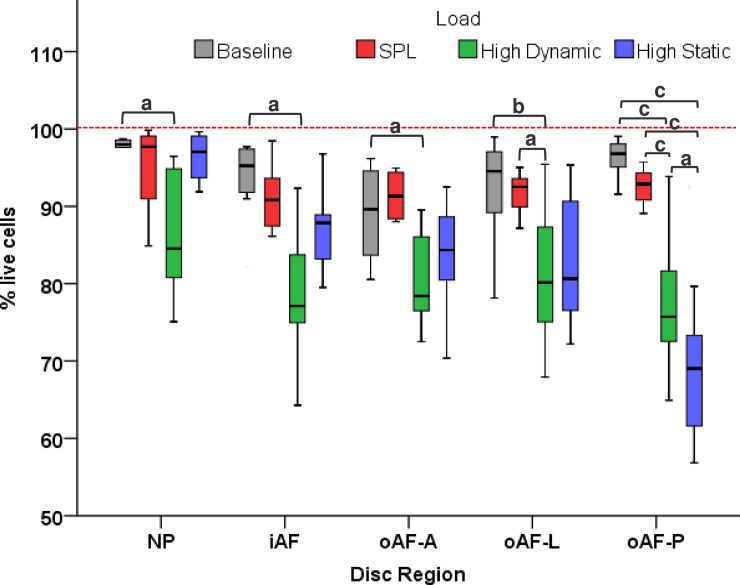
Boxplot graph of cell viability. The graph shows cell viability (mean percentage live cells ± SD) at baseline (day 0; gray boxplots) and after the 14 day culture period with respectively SPL load (red), high dynamic load (green) and high static load (blue). The cell viability data is split on the x-axis over the five distinct IVD regions; nucleus (NP), inner annulus (iAF), and the outer annulus (oAF, anterior; lateral and posterior). Brackets indicate significant statistical differences between groups when comparing in a linear mixed model with Bonferroni post-hoc testing: *P* values are indicated by: ^a^*p*<0.05; ^b^*p*<0.01; ^c^*p*<0.001.

In Fig 6, the cell viability percentages from the CTG/PI staining are shown in more detail in a boxplot graph. At baseline and after 14 days of culture and loading cell viability (percentage live cells) is shown per IVD region per experimental group. Baseline values correspond with previously observed values [30] and in the SPL control group we don’t observe any significant decreases in cell viability after 14 days of culture and loading when comparing to baseline. As indicated by the bars in the graph (Fig 6), high dynamic loading is detrimental for the nucleus and inner-annulus as well as all three outer annulus regions, with statistically significant decreases of cell viability in 14-days. Most importantly, in the high static loading group we observe a strong drop in cell viability almost exclusively in the posterior outer annulus (69.2±9.2), whereas the cell viability in the nucleus remains at baseline and SPL levels (96.4±3.5).

**Fig 7:**
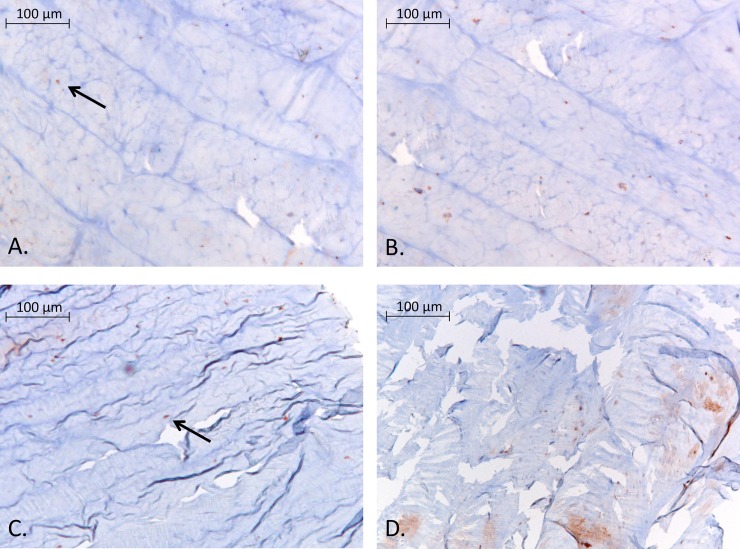
Immunostainings for cleaved caspase 3 on annulus regions. Representative images of apoptosis staining using a cleaved caspase 3 antibody with haematoxylin counter staining. Fig 7 a and b showing detailed images (20x magnification) of the anterior annulus region of respectively an SPL (left) and an HS (right) sample. Fig 7 c and d are from the posterior annulus region of the same SPL (left) and HS (right) discs. Exemplary apoptotic bodies are marked by arrows in the SPL images a and c, in images b and d they are abundantly present so no markings were added.

On the Caspase-3 immunostained sections all experimental groups had some positive stainings for apoptotic bodies in all disc regions (Fig 7). The SPL samples had the least positive staining and between the overload groups there was no clear difference. Nor could we observe a regional pattern in the amount of apoptotic bodies (Fig 7A and 7B versus 7C and 7D) in any group. The ratio between Caspase-3 positive stained cells and the total haematoxylin stained cells was approximately 1:20 for SPL (Fig 7A and 7C) and 1:5 for both HS (Fig 7B and 7D) and HD (not shown).

### Histology after culture

**Fig 8:**
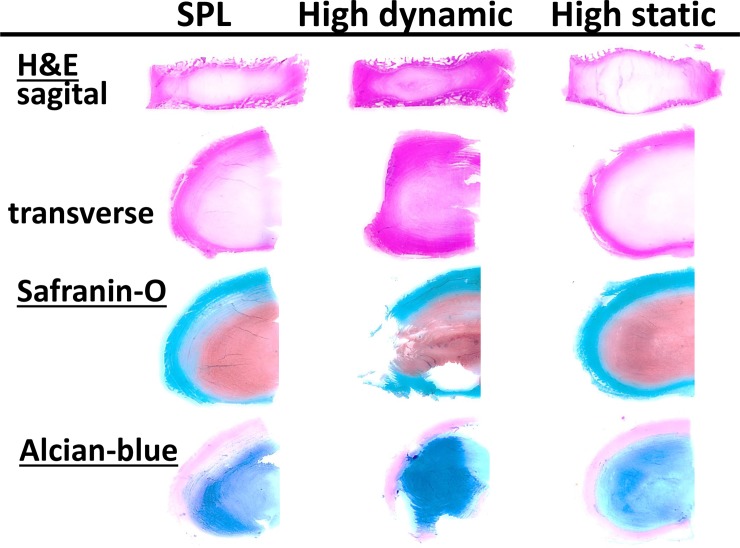
Histological scoring of IVD degeneration. This overview shows representative images of histological sections from the three experimental groups after the 14-day culture and loading experiment as used for the scoring of IVD degeneration according to the Rutges scale. From top to bottom, section are: midsagittal H&E stained sections to score endplate damage (left posterior, right anterior); transverse H&E stained sections to score annulus and nucleus matrix morphology (all left haf IVDs: above anterior; left lateral; bottom posterior); transverse sections stained with Safranin-O and Alcian-blue to observe changes in proteoglycan and GAG distribution. All sections and stainings combined depict the characteristics of the transition zone. For the specific scores of each experimental group per section and a description of the observed degenerative changes due to culture and loading, please see Table 2 and the related results paragraph.

The 14-day overloaded IVDs were difficult to cut and when comparing the high dynamic and static slides with the SPL slides, we can directly observe that the quality in the overloaded groups is poor, especially for the transverse sections (Fig 8). The samples of the overload groups are atypical examples of (more or less) intact transverse slides of a dynamically (middle column) and statically (left column) overloaded IVD. These intact samples demonstrate the degenerative changes (i.e. deformation in the radial plane and changes in matrix structure) most clearly and were used for the Rutges classification.

**Table 2:** Mean histological scoring according to the Rutges scale.

	Endplate	Morph. AF	Boundary AF & NP	Cellularity NP (CTG)	NP morph. (H&E/AB)	NP staining (Safr-O/AB)	total score
SPL	0	0	0	0	0	0	0
HD	2	2	1	1	2	1	9
HS	1	1–2	1	1	0–1	1	5–8

Table 2. Descriptive histological scores (means per group) of IVD degeneration using the Rutges classification. Zero represents no degenerative changes; 1 mild/moderate degenerative characteristics and 2 severe degeneration. In the continues scale a total score of 0 represents a completely healthy disc and the maximum score of 12 being completely degenerated.

On the H&E stained sections of the dynamically overloaded discs, the outer annulus showed fairly random patterns of matrix disruption (Fig 8, middle panel). On the sagittal section these disruptions occurred mostly in the anterior annulus and through the endplates, on transverse sections mostly in the anterior and lateral region (endplate and annulus morphology Rutges grade 2 “severe” degeneration). Interestingly, before sectioning IVDs appeared intact macroscopically, although sectioning revealed weakening of the anterior and lateral regions. The effect of height loss with high dynamic loading can also be observed by the more dense appearance and disruption of the nucleus and annulus matrix (Fig 8, middle panel Safranin-O and Alcian-blue staining; NP morphology Rutges grade 2 “severe” degeneration; NP staining grade 1 “mild” change) and the migration of nucleus tissue towards the periphery of the disc in both anterior and posterior direction (Fig 8, middle panel H&E sections; AF boundary grade 1 “moderate” degeneration). The outcomes of the Rutges scorings are summarized in Table 2.

On all sections of the statically overloaded IVDs we can observe a lighter staining and swelling of the nucleus (Fig 8, right panel). On the sagittal H&E section the endplate bulges around the nucleus with local thinning, but is largely intact (grade 1 “mild” degenerative change). This effect can also be observed for the outer annulus on the transverse H&E sections, with the matrix on most slides overall appearing more dense and thin. However, in a third of the IVD specimens the posterolateral and posterior region of the disc was so thin and fragile, that it was not possible to preserve it with sectioning (grade 1–2, “moderate” or “severe” changes). In the H&E and Alcian-blue sections the nucleus and annulus can still be distinguished, but the transition zone can no longer be identified (grade 1 “moderate” degenerative change). The transverse Safranin-O and Alcian-Blue sections show that the matrix structure of the nucleus appears fairly homogeneous (grade 1 “mild” degenerative changes), with only slightly less intense staining (grade 1 “mild” degenerative change).

**Fig 9:**
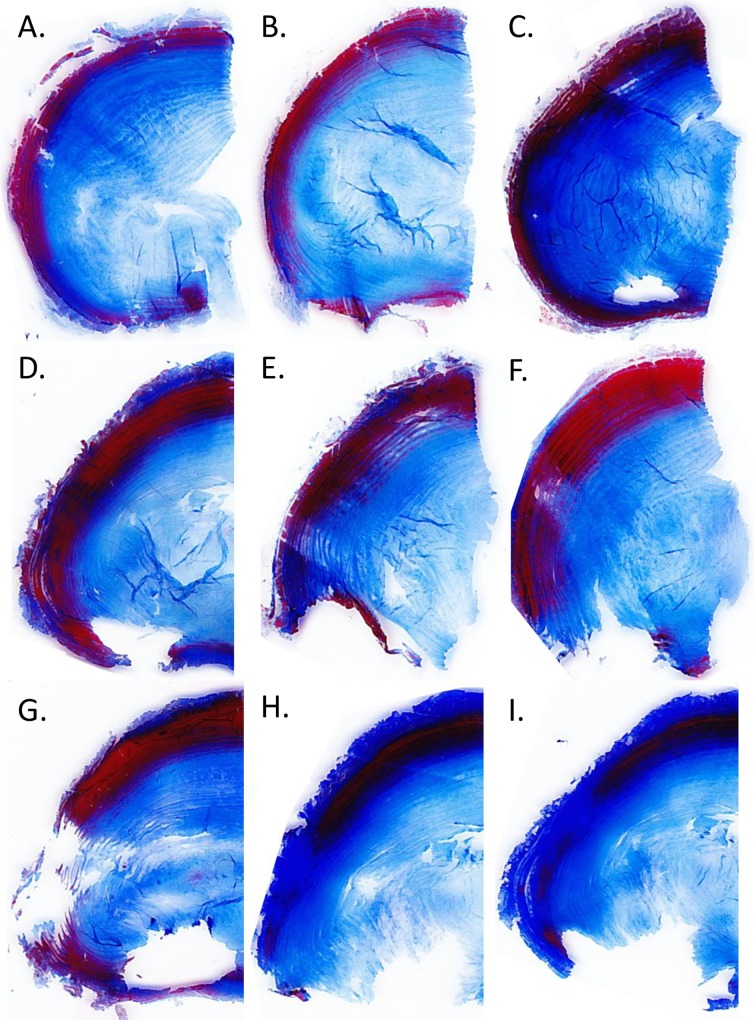
Damage pattern of static overloading. Panel of nine exemplary images of transverse sections of IVDs overloaded with static loading. The Masson’s Trichrome staining depicts were the matrix has a porous structure due to a higher type 1 to 2 collagen ratio and low proteoglycan content (stained red: anterior and lateral outer annulus), were the collagen ratio is in favour of collagen 2 to 1 and overall more abundant than PG’s (dark blue: inner annulus) and were mostly PG’s make up the IVD matrix and some collagen type 2 (light blue: nucleus). Not a single disc showed macroscopic signs of herniation or bulging when taken out of the LDCS after 14 days of culture and overloading with static axial load. Starting in the upper left corner, image A shows only mild displacement of especially the inner annulus lamellae in the posterolateral corner, with displacement and disruption of the inner- and outer annulus being progressively worse from image B to I. These images provides an overview of the pattern of damage inflicted by the static axial overloading on the posterolateral matrix of the discs as revealed after histological sectioning.

In contrast to the intact overloaded samples shown in Fig 8, in Fig 9 a more representative series of images is shown of transverse sections of statically loaded IVDs. Image A through I show progressive degenerative changes (as the large range in the Rutges scores for statically loaded specimens quantified in Table 2). Shown by this panel of images, regardless of the degenerative state of the IVDs, the posterolateral corner always sustained some damage, whether it is a mild displacement of inner annulus lamellae (image A) or complete disruption of inner- and outer annulus (image I). This is in contrast to the dynamically overloaded IVDs where defects occurred in all regions of the disc on transverse sections (Fig 8).

## Discussion

In the current study we show that with prolonged axial overloading of caprine lumbar IVD; 1) significant height loss occurs without changes in the exterior pressure distribution over the disc, 2) general cell death and matrix disruption occurs in all disc regions with high dynamic overloading and 3) static overloading results in a posterior annulus region specific breakdown, with significant cell death and matrix disintegration and relative sparing of the nucleus region. Therefore, we conclude that the manner (dynamic or static) of axial loading influences the effect on the various regions and structures of the IVD differently and prolonged static axial overloading primes the lumbar caprine IVD for posterolateral herniation.

We conducted these series of experiments to find an explanation for the clinically observed posterolateral predilection of hernia’s in the human lumbar spine. This could be regarded as counter-intuitive when taking into account that in the *in vivo* situation, due to the lumbar lordosis, affiliated flexion in the motion-segments with wedging of the IVD and the concomitant loading of the posterior elements of the spine, together would attribute to an anterior predilection from a strictly mechanical stand point [37,38]. However, as we show in our ex vivo model loading a complete lumbar IVD with strictly axial static overloading, a region specific mechanically induced degenerative cycle [39] is triggered specifically in the posterior region of the annulus.

Various studies have shown the mechanical [33,40–42] and biological similarities [32,43,44] between human and caprine goats. Anatomical studies by Eyre *et al*. and Inoue *et al*. provide detailed description of the differences between the anterior and posterior annulus in various animal and human IVDs. Notable similarity of the region specific characteristics are the variations in collagen ratio’s (more type 1 versus type 2 anteriorly) [45,46], bundle thickness and orientation of the annulus lamellae [44,45]. The findings in our current study on the histological sections are in accordance to these findings (Figs 2, 8 and 9) [47,48]. Also, in a study on human lumbar IVDs by Iatridis *et al*. investigators showed that both water and GAG concentration varied between regions, with lowest values for GAG and water in the anterior and lateral regions of the outer annulus and highest values in the centre of the nucleus [49]. We observe the same pattern in distribution in our lumbar caprine IVDs, confirming the resemblance of caprine lumbar IVDs to human IVDs (Fig 2) [45,50]. We therefore believe the observed effects of the overloading on the caprine IVDs are representative for the human situation.

Whether the described structural differences between the annulus sections (lamellae structure, collagen composition and GAG distribution) are genetically predisposed, the result of natural ageing and adaption to the regionally different biomechanics, caused by the detrimental effects of overloading, or the cumulative result of all, remains a question. However, by eliminating confounding factors in the LDCS model and simply comparing the effects of strictly axially applied dynamic versus static overloading to lumbar caprine IVDs, we can conclude that the manner of overloading affects the nucleus, anterior and posterior annulus differently.

In cadaver studies on human lumbar motion-segments [14,51] and ovine lumbar discs it was found [52] that discs would only herniate with the combination of flexion and high (near failure) compressive force. However, in the current study we show that even within near physiological ranges (Fig 1) of axial load (without inflicting direct traumatic mechanical damage to the disc), static overloading over a prolonged period of time already primes the IVD at a cellular and matrix level for a posterior herniation, providing an explanation for the observation that most herniations in practice occur during seemingly arbitrary mechanical conditions.

During the 14-day culture period, both dynamically and statically overloaded IVDs lost a similar amount of height, which was significantly more than the simulated-physiologically loaded control (on average 1,0 mm; Fig 3). The height loss of the IVDs in response to axial overloading was not load or disc region specific, i.e. both dynamic and static overloading inflicted the same height loss, equally in the anterior and posterior region of the IVD. Despite the inflicted height loss and the difference in structure of the anterior and posterior outer annulus, the transfer of the axial load through the IVD did not change significantly over time in any region (Fig 4). Vergroesen *et al*. already showed that intradiscal pressure in caprine lumbar IVDs is correlated to loading history and height loss. The incessantly even exterior distribution of pressure over the endplate shows that even with significant height loss, the biomechanical properties of the IVD remain largely intact [33]. However, McNally and Adams *et al*. showed with intradiscal pressure transducer that internal stresses differ between disc regions depending on the type of load applied and degenerative state of the IVD [17,19]. Interestingly, the bulk height loss in the current study had already occurred after 4 days of overloading and the pressure in the static group remained slightly higher in the posterior region during the entire culture period. We can therefore speculate that with static overloading, these differences caused slightly higher internal stresses posterolaterally, biologically significant enough to inflict the observed higher amount of cell death and matrix breakdown in this region.

Fluorescent PI stained cells are more abundant and region specific compared to the caspase-3 positive stained cells in the overloaded groups. Therefore, we conclude that the additional loss in cell viability in the posterolateral region with static overloading is not restricted to cell apoptosis alone, but–at least in part- also due to cell necrosis or autophagy. This is in accordance with others reports showing high inflammatory and necrotic activity with mechanical overloading and degeneration [30,53–56]. This is a particularly important finding in the light future targeted therapeutics, as it shows that implication of an agent with a broad-spectrum anti-inflammatory function is more likely to succeed, than a very specific single-target agent.

Traditionally, the IVD response to loading is studied by distinguishing three regions in the IVD: nucleus, inner- and outer-annulus. To our knowledge, this is the first study to map the differences between the anterior, lateral and posterior regions of the disc in response to overloading during culture (Figs 5 and 6). Therefore, it is difficult to correlate the observed patterns in cell viability across the IVD to other published work on the matter. However, the found results for the nucleus, inner- and outer-annulus concur with our earlier studies on the effect of high dynamic and static overloading on the caprine lumbar disc (Fig 6) [30]. Also, many studies have analyzed regional matrix remodeling effects due to dynamic versus static overloading and found similar patterns of degenerative changes. For example, Korecki *et al*. in 2008 studied the response to different magnitudes of dynamic load in different IVD regions. They also observed that with dynamic overloading there was remodeling behavior in the nucleus (up-regulation of collagen type 1 and MMP3 activity), whereas in their static control group there was no response [26]. Furthermore, Alkhatib et al. reported significant cell death with up regulation of inflammatory gene expression (interleukins) and associated loss of proteoglycan after high magnitude loading, which in turn caused significant neurite sprouting in PC12 cells known to be linked to neo-innervation in IVDs and discogenic pain [56].

When comparing the histological sections of the overloaded discs with the SPL specimens (Fig 8), we find clear evidence for degenerative changes in both nucleus and annulus regions with dynamic overloading. We analyzed our findings using the validated histological scoring system of Rutges [36]. This new scoring system is an adaptation of the older Boos score and preferred to e.g. the Thompson scale, as it is easy to use and understand even for the non-expert observer and still provides detailed and semi-quantitative scoring of degenerative changes [3,36,57]. The changes due to overloading observed on the histological sections are similar to those described in our earlier publications [29,30] and the application of the Rutges scale provides a reproducible description of the degree of degeneration (Table 2).

A limitation of the use of the Rutges scale for the purpose in current study is that it does not distinguish between the anterior, lateral and posterior regions and the gross (region specific) damage of overloading as seen in the majority of sections cannot be accounted for. Therefore, especially when assessing the degenerative changes on the statically overloaded IVDs, we found large variation in the observed damage to the annulus region (Table 2), due to the finding that the anterior outer annulus was less affected when compared to the posterior annulus as depicted in the Masson’s Trichrome stained sections in Fig 9.

Another limitation of the current study is that the histological sections and the applied score, do not provide quantitative data on matrix content. However, we feel that any attempt to quantify these possible (small) changes over the culture period by, for instance quantitative biochemical assays, would most likely depict the inter-IVD and regional variance without reliably measuring effects due to overloading. Future studies should include a non-disruptive quantitative way of measuring matrix components (e.g. quantitative MRI) to reliably measure the specimens at baseline and after the intervention.

Thus, the most important finding of the current study is that although the mechanical changes due to overloading of the disc are the same (significant height loss without change in pressure distribution with both HD and HS), the manner of overloading–dynamic versus static- will affect the biological response within the disc and is region specific. The strains in the disc caused by static axial overloading will firstly affect the posterolateral region. The nucleus is relatively spared, staying hydrated and pressurized, while the posterior region by comparison is weakened by apoptotic and necrotic cell response which will trigger the vicious cycle of degeneration [39]. Our finding provide a clear biological rational for the observed predilection of hernia’s in the posterolateral corner of the lumbar spine.

We can speculate this might be at the basis of low back pain and herniation epidemic among the population leading a sedentary and sitting life style [10,58,59], which is predominately static axial load on the back. In practice, occupational health programs aimed at prevention of low back pain and hernia’s in the workforce should focus on prevention of static overloading on the lumbar spine due to i.e. prolonged sitting in a single posture. In diagnostics of DDD, especially when spinal overloading is suspected, the posterior region of the lumbar IVD might be the preferred site to detect early degenerative changes. Also, the region specific degenerative mechanisms within the disc should be taken into account in the design and implementation of preventive and curative therapies against lumbar disc degeneration and herniation. For instance, one should intervene early in the degenerative cascade with therapeutics that hamper cell driven matrix breakdown, and avoid the posterior region when injectable therapeutics are applied [60]. In addition, our study provides new evidence to underline the conclusion by Bron *et al*. [61], Gregory *et al*. [62] and Iatridis *et al*. [63], that clinical treatment of DDD and LDH should not only target rehydration or regrowth of the nucleus pulposus, but should also target repair and strengthening of the (posterior) outer annulus to confine the (regenerating) nucleus.

Whether the posterolateral part of the lumbar IVD is by concept the weakest mechanical site or if this weakness is an attainment of a regional degenerative cell response to overloading, cannot be deduced. The impact of changes due to natural ageing, adaption during life to the mechanical environment and regional specific degenerative changes within the disc in the development of DDD remains subject to further study.
